# Global Perspective on Acute Kidney Injury in Chile

**DOI:** 10.34067/KID.0000000798

**Published:** 2025-04-02

**Authors:** Cristian Pedreros-Rosales, Gonzalo Ramírez-Guerrero, Ignacio Gacitúa-Meneses

**Affiliations:** 1 Departamento de Medicina Interna, Facultad de Medicina, Universidad de Concepción, Concepción, Chile; 2 Nephrology Service, Hospital Las Higueras, Talcahuano, Chile; 3 Nephrology and Dialysis Unit, Carlos Van Buren Hospital, Valparaíso, Chile; 4 Sección de Nefrología, Hospital Clinico de la Universidad de Chile, Universidad de Chile, Santiago, Chile; 5 Nephrology Service, Hospital Barros Luco–Trudeau, Santiago, Chile

**Keywords:** acute kidney failure, AKI, acute renal failure, renal replacement therapy

## Introduction

**Figure 1:**
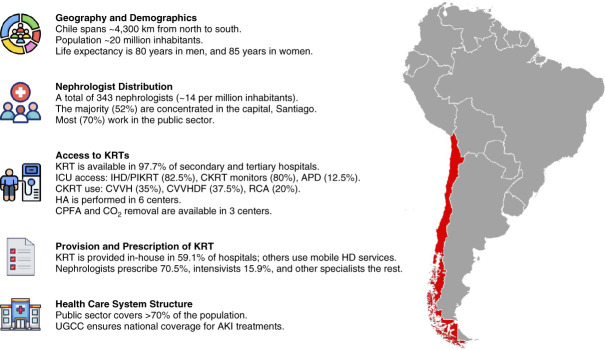
**Renal health care landscape for AKI management in Chile.** Chile spans approximately 4300 km, creating geographic and health care access challenges. Figure highlights nephrologist distribution, concentrating in Santiago and regional disparities. It details KRT availability, including intermittent and continuous modalities, peritoneal dialysis, and advanced extracorporeal techniques. In addition, it outlines KRT prescription roles among specialists and the UGCC's role in national patient transfers. Chilean health system operates under a dual public-private model, with the public sector covering over 70% of the population and ensuring nationwide AKI treatment access. APD, acute peritoneal dialysis; CKRT, continuous KRT; CPFA, coupled plasma filtration adsorption; CVVH, continuous veno-venous hemofiltration; CVVHDF, continuous veno-venous hemodiafiltration; HA, hemoadsorption; HD, hemodialysis; ICU, intensive care unit; IHD, intermittent hemodialysis; PIKRT, prolonged intermittent KRT; RCA, regional citrate anticoagulation; UGCC, Centralized Bed Management Unit.

Chile extends from the Atacama Desert to the Patagonian glaciers (Figure 1). Its dual public-private health system, the Explicit Health Guarantee,^1,2^ guarantees access to 87 priority conditions, including kidney diseases.

AKI remains a significant challenge in Chile, with gaps in epidemiologic monitoring, early diagnosis, and equitable access to treatment.

This review provides a focused analysis of AKI in Chile, identifying key gaps in surveillance, workforce distribution, and therapy access while discussing health care system challenges and policy responses.

## Epidemiology of AKI in Chile

The history of KRT in Chile dates back to 1957 when San Borja Hospital in Santiago performed the first hemodialysis on a patient with septicemia using a Kolff-Brigham artificial kidney.

AKI epidemiology has evolved alongside public health transitions. In the late 20th century, septic abortion was a leading cause of AKI, but declined with family planning policies and improved obstetric care.^3^ Since the 1980s, renal therapy expanded across hospitals to address septic shock, ischemia, and nephrotoxins. In 1990, continuous KRT (CKRT) was introduced at university hospitals, complementing intermittent hemodialysis (IHD).^4^

A national survey across 38 hospitals confirmed broad KRT availability but with significant disparities. IHD and prolonged intermittent KRT (PIKRT) are the most common techniques, whereas CKRT use varies. In some hospitals, dialysis unit teams manage KRT; in others, intensive care unit (ICU) teams do. Some centers use mixed models.

Chile has 343 nephrologists (14 per million inhabitants), with 52% concentrated in Santiago (Figure 1), among them 59 pediatric nephrologists. Nephrology training requires 3 years of internal medicine residency and a 2-year subspecialty program. Although no national data exist on the average age of nephrologists, expanding training programs suggests a growing interest in the field.

Like Argentina, Peru, Ecuador, and Bolivia, Chile lacks a national AKI registry, relying instead on hospital-based studies and surveys.^5^ However, unlike these countries, Chile recently conducted a nationwide survey with the Ministry of Health and the Chilean Society of Nephrology, providing a structured assessment of KRT availability and regional disparities.

Regional inequalities in AKI care are common in Latin America. Argentina has better AKI care access in central provinces, while Peru and Ecuador concentrate AKI services in major cities, leaving rural areas underserved. Bolivia and Ecuador have fewer than 16 nephrologists per million inhabitants.^5^ Despite a higher nephrologist-to-population ratio, Chile still faces workforce distribution challenges and limited access to advanced therapies in remote regions.

## Etiology and Risk Factors

AKI in Chile has a multifactorial etiology, similar to other Latin American countries. Sepsis remains the leading cause, particularly in ICU patients, followed by nephrotoxicity from non-steroidal anti-inflammatory drug, aminoglycosides, and contrast media. Other contributors include rhabdomyolysis (trauma-related), perioperative AKI (especially in cardiac surgery), and hypovolemia due to severe dehydration in non-ICU patients.^6–8^

Chile's aging population significantly influences AKI patterns. Between 1950 and 2017, the population aged 60 years or older increased 6.8-fold, comprising 16.2% of the total. This trend is most pronounced in urban areas, where hypertension and diabetes—key AKI risk factors—are highly prevalent.^9^

Chile's AKI risk factors largely mirror those in Argentina and Peru, where sepsis, nephrotoxicity, and postoperative complications predominate. However, Bolivia and Ecuador face additional burdens from endemic infections, including leptospirosis, malaria, and dengue, which significantly contribute to AKI incidence. Unlike these countries, Chile has a lower burden of infectious disease-related AKI but a higher prevalence of multimorbidity-related AKI in an aging population.

## Diagnosis and Management of AKI

AKI diagnosis in Chile follows Kidney Disease Improving Global Outcomes guidelines, although full implementation remains challenging, particularly outside major urban centers. A key limitation is the restricted use of advanced biomarkers such as cystatin C, neutrophil gelatinase-associated lipocalin, or urinary (tissue inhibitor of metalloproteinases-2)×(insulin-like growth factor-binding protein 7), which are available mainly in academic hospitals or research settings, limiting their role in routine early diagnosis.

KRT is widely available in major hospitals, but extreme regions face barriers related to infrastructure and staffing. Most ICUs provide KRT, yet access to CKRT, acute peritoneal dialysis, and regional citrate anticoagulation remains limited (Figure 1).

Nephrologists prescribe 80%–85% of IHD/PIKRT therapies, but for CKRT, their involvement decreases to 55%, with intensivists covering the rest. ICU nurses primarily manage CKRT, while specialized in-hospital dialysis nurses handle IHD/PIKRT. Some institutions use mixed models, where both teams collaborate.

## Challenges of the Health System in the Management of AKI

Chile's health system operates under a dual public-private model (Fondo Nacional de Salud/Instituciones de Salud Previsional), with the public sector covering over 70% of the population.^1^ The Centralized Bed Management Unit (UGCC) plays a pivotal role in ensuring that critical therapies are available nationwide, even during periods of high demand, by coordinating patient transfers and optimizing KRT access.^9,10^

Advanced KRT resources remain concentrated in urban hospitals, while remote regions face infrastructure and specialist shortages, limiting AKI care. With better access to advanced diagnostics, the private sector serves a smaller population, and its services depend on financial capacity or health plans. In extreme regions, complex therapies rely on public hospitals, strengthened by UGCC coordination.

## Economic Effect of AKI in Chile

AKI imposes a substantial financial burden. Direct costs include prolonged ICU stays, mechanical ventilation, and higher mortality, increasing expenses for KRT and specialized treatments.^7^ Although Fondo Nacional de Salud covers most public-sector costs, delayed access to advanced therapies often leads to complications and higher expenditures.

Indirect costs, such as transportation, income loss, and postdischarge care, exacerbate social and economic inequalities. The UGCC uses Diagnosis-Related Groups agreements to optimize spending, ensuring cost-effective case resolution in private providers. In addition, national initiatives aim to streamline public KRT costs and reduce reliance on external services.

## Challenges and Conclusions

AKI remains a major challenge, exposing inequities in advanced therapy access and the need for improved care in underserved regions. Infrastructure gaps and nephrologist shortages in remote areas limit equitable AKI treatment, emphasizing the need for targeted investments and workforce expansion. Economic sustainability is also a priority, requiring cost-effective resource allocation to maintain high-quality care. Strengthening the UGCC is crucial to improving referral efficiency, particularly in regions with limited resources.

While Kidney Disease Improving Global Outcomes guidelines serve as a reference, critical gaps persist in biomarker implementation, collaborative nephrology-intensivist models, and specialized training in peripheral regions. The public sector, supported by the UGCC, is essential for complex therapies but faces infrastructure, staffing, and financial limitations that must be addressed to improve AKI outcomes.

Better care integration, medical training, and early diagnosis are key to equitable AKI management. A national AKI registry and electronic alert systems are vital to bridging current gaps, alongside expanding access to advanced diagnostics and therapies, particularly in underserved areas.

Chile has successfully implemented CKD prevention, telemedicine, and early detection strategies. Adapting these frameworks for AKI could enhance risk stratification, early intervention, and coordinated care pathways. The coronavirus disease 2019 pandemic further emphasized the need for robust AKI management, as surges in severe cases increased ICU demand for KRT. Lessons from this crisis highlight the urgency of national policies to strengthen AKI prevention, early detection, and specialized care.

The Chilean Society of Nephrology calls for a unified intersectoral effort, led by the Ministry of Health and supported by medical societies, to position AKI management as a public health priority. This initiative must strengthen infrastructure, expand training, and integrate advanced technologies to ensure equitable, high-quality care nationwide.
